# Transport properties of B-site codoped CaHfO_3_ proton conductors with octahedral distortion

**DOI:** 10.1039/d4ra06213b

**Published:** 2024-11-18

**Authors:** Wenlong Huang, Mingze Lv, Ying Li, Yushi Ding, Jiayao Lu, Chunsheng Zhuang, Pengfei Yue, Wei Zhang

**Affiliations:** a School of Metallurgy, Northeastern University China liying@mail.neu.edu.cn dingysh@smm.neu.edu.cn; b Liaoning Key Laboratory for Metallurgical Sensor Materials and Technology, Northeastern University China; c Institute of Applied Physics, Henan Academy of Sciences Zhengzhou Henan 450008 China

## Abstract

Perovskite-type solid electrolytes exhibit a diverse range of conductive properties due to the competition and coupling of multiple degrees of freedom. In perovskite structures, B-site and X-site ions form topological octahedral sublattices, which are instrumental in regulating transport properties for various charge carriers. However, research focused on the relationship between octahedral distortion and conductive properties in perovskite-type proton conductors remains limited. In this study, dopants such as Ge, Sn, Pr, and Ce were selected to modify the degree of BO_6_ octahedral distortion in CaHf_0.9_Sc_0.1_O_3−*δ*_. The relationships between conductivity, transport number, mobility, and the distortion degree were systematically investigated. The data indicate that both proton and oxygen ion mobilities initially increase with the octahedral distortion angle and then decrease, and CaHf_0.8_Sn_0.1_Sc_0.1_O_3−*δ*_ with an octahedral distortion angle of 15.6°, exhibited the highest ionic mobilities and conductivities. The BO_6_ octahedral distortion appears to limit oxide ion conduction while enhancing the proton transport number. However, excessive doping generates additional oxygen vacancies, which adversely affect proton conduction. Under the combined influence of these factors, CaHf_0.8_Ce_0.1_Sc_0.1_O_3−*δ*_ achieved the highest proton transport number of 0.503 at 800 °C. Overall, this work provides insights into the relationship between octahedral distortion and conductive properties, suggesting that co-doping is a feasible approach for further regulating carrier mobility properties.

## Abstract 2

Both proton and oxygen ion mobility initially increase with octahedral distortion angle and then decrease, and CaHf_0.8_Sn_0.1_Sc_0.1_O_3−*δ*_ with the octahedral distortion angle of 15.6° exhibited the highest ionic mobilities and conductivities.

## Graphical abstract

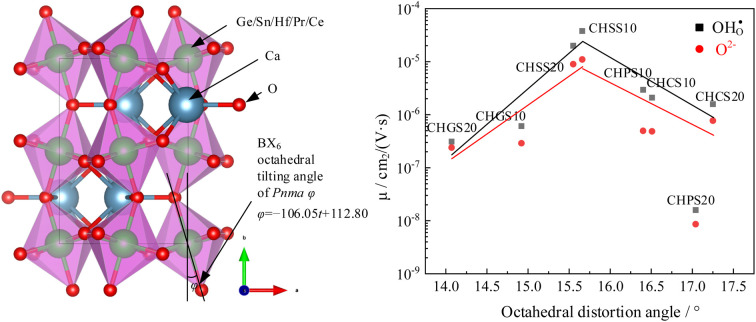

## 1. Introduction

ABO_3_ perovskite-type proton conductors have been widely investigated due to their potential applications in fuel cells,^1–4^ electrochemical sensors,^5,6^ and electrochemical synthesis.^7,8^ Under operating conditions, these proton conductors simultaneously exhibit proton, oxide ion, and hole conduction. The sensing electromotive force (EMF) signal of a hydrogen sensor can be described by eqn (1):1
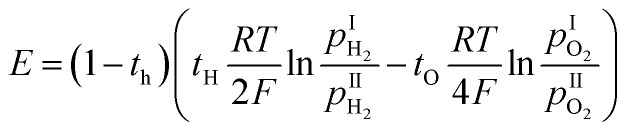
where *t*_H_, *t*_O_, and *t*_h_ represent transport numbers of proton, oxide ion, and hole, respectively. It is important to note that the conduction of oxide ions and holes can introduce significant errors in measured electromotive force. Therefore, to ensure greater accuracy, the proportion of proton conductivity relative to the total conductivity, expressed as the proton transport number, should approach 1.

Bonanos *et al.*^9^ reported that the orthorhombic distortion of perovskites can reduce oxide ion conductivity without significantly affecting protonic conductivity. Consequently, CaZrO_3_-type and CaHfO_3_-type proton conductors with orthorhombic distortion, particularly CaZr_0.9_Sc_0.1_O_3−*δ*_ and CaHf_0.9_Sc_0.1_O_3−*δ*_, have shown the highest protonic transport numbers across various atmospheres.^10–13^ This phenomenon can be explained as follows:

In perovskite-type proton conductors, protonic migration primarily occurs *via* a rotation-jump mechanism. The energy barrier for this process is predominantly determined by the jump mechanism, with the jump barrier exhibiting a strong linear relationship with the distance between the proton and the second nearest oxygen (H–O).^14^ Similarly, the energy barrier for oxide ionic migration is influenced by the analogous O–O pathway.^15^ As a result, high protonic conductivity is often accompanied by high oxide ionic conductivity. Specifically, the tilting of the BO_6_ octahedra extends both the H–O and O–O distances.^9,16^ Given that oxide ions have a greater mass than protons, their transfer is slower under the same conditions. Therefore, the tilting of the BO_6_ octahedra hinders oxide ion movement more than proton movement, thereby increasing the protonic transport number at the cost of reduced overall conductivity.

Based on this mechanism, two methods for enhancing the protonic transport number can be readily inferred: (1) increasing the dopant element content M in AB_1−*x*_M_*x*_O_3−*δ*_, and (2) designing proton-conducting matrix materials with greater orthorhombic distortion, such as CaCeO_3_ and CaPrO_3_. However, at present, neither method achieves the desired results. In the first approach, increasing the concentration of trivalent dopant ions results in the formation of a significant number of oxygen vacancies in the perovskite structure, which leads to defect clustering and consequently reduces the conductivity of the proton conductor. In the second approach, the high degree of distortion in CaCeO_3_ and CaPrO_3_ exceeds the structural tolerance of perovskites, rendering their synthesis *via* conventional methods currently infeasible.

In our previous studies,^17^ we selected CaHf_0.9_Sc_0.1_O_3−*δ*_, which possesses a high protonic transport number, as the base material and utilized Ce, with its larger ionic radius compared to Hf, as a co-dopant to further enhance the proton transport number. This approach suggests that it is feasible to increase the orthorhombic distortion of the perovskite structure and thereby improve the proton transport number by co-doping with tetravalent elements without increasing oxygen vacancies. Nevertheless, we consider our prior research to be a preliminary exploration; thus, there remains a need to design a series of perovskites with varying degrees of distortion to establish a quantitative relationship between distortion degree and conductivity.

In this study, tetravalent dopants Sn, Ge, Pr, and Ce were selected to construct perovskites with varying degrees of distortion while minimizing changes to the oxygen vacancy concentration. Perovskites of the form CaHf_0.9−*x*_M_*x*_Sc_0.1_O_3−*δ*_ (M = Ge, Sn, Pr, and Ce; *x* = 0.1, 0.2) were prepared using the solid-state reaction sintering process. The valences of the dopant elements were determined by X-ray photoelectron spectroscopy (XPS). The conductivities of the resulting ceramics were measured at temperatures ranging from 400 °C to 800 °C using AC impedance spectroscopy. The partial conductivities and transport numbers of proton, oxide ion, and hole were analyzed through the defect equilibria model. The effects of dopant element and concentration on conductivities, ionic mobilities, and transport numbers were clarified. Additionally, a straightforward method for calculating the distortion angle of the BO_6_ octahedron based on the chemical formula was proposed. Common relationships between the distortion of the BO_6_ octahedron, the valence reduction tendencies, and the transport numbers and mobilities were clarified.

## 2. Experimental

CaHf_0.9−*x*_M_*x*_Sc_0.1_O_3−*δ*_ (CHMS) (M = Ge, Sn, Pr, Ce, *x* = 0.1, 0.2) (CHGS, CHSS, CHPS, and CHCS) proton conductors were prepared through solid state reaction sintering process using CaCO_3_(AR), HfO_2_(3N, Zr < 0.5%), Sc_2_O_3_(3N), GeO_2_(5N), SnO_2_(2N5), Pr_6_O_11_(3N), and CeO_2_(3N). Briefly, stoichiometric mixtures of chemical reagents were initially dispersed in ethanol and subjected to ball milling for 10 h. Following solvent evaporation, the dried powders were pressed into cylindrical columns (*φ* 25 mm × *d* 15 mm) and calcined at 1400 °C in air for 10 h. The powders, ground from these columns, were then pressed into disks (*φ* 10 mm × *d* 2 mm) using cold isostatic pressing at 200 MPa and subsequently sintered at 1600 °C in air for 10 h.

The crystal structures of the as-obtained CHMSs were characterized by X-ray diffraction (XRD, Rigaku Ultima IV), equipped with Cu Kα radiations (40 kV and 30 mA) in the 2*θ* range of 10–90°. The tolerance factors were calculated by eqn (2) using the Shannon ionic radii data.^18^2
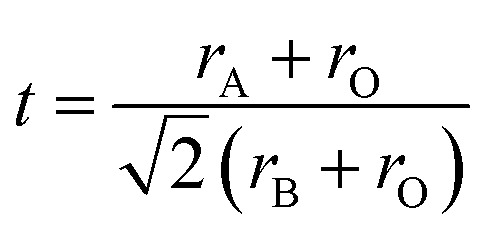
where *r*_A_, *r*_B_, and *r*_O_ represent the A-site, B-site, and oxide ion radii of ABO_3_, respectively.^19^

The micro-morphologies of the ceramic specimens were observed by field emission scanning electron microscopy (FESEM, SU8010). The XPS spectra were recorded by ESCALAB 250Xi with a monochromatic Al/Mg binode X-ray source. The conductivities were determined by electrochemical impedance spectroscopy (EIS). To this end, both sides of sintered ceramic disks were first polished to ensure precise electrode area.

The surfaces were subsequently coated with Pt paste, connected with Pt electrode wires, and calcined at 900 °C for 30 min in air to create porous electrodes. A mixed atmosphere containing various partial pressures of oxygen and water vapor was flown through the samples at the rate of 100 mL min^−1^. The oxygen partial pressure was controlled using a mass flow controller, mixing O_2_ (99.9%), O_2_/Ar (5.00%), and Ar (99.9%). The water vapor partial pressure was controlled by evaporating quantitative water in a pre-furnace setup, while the water flow was controlled by a peristaltic pump with a 0.5 mm silicone tube.

The impedance spectroscopy of the samples was recorded by a frequency response analyzer (Solartron 1260, voltage 500 mV, measurement frequency 1 Hz to 5 MHz).

The partial conductivities of protons, oxide ions, and holes were analyzed by defect equilibria model,^20–22^ which can be expressed by eqn (3) and (4):3
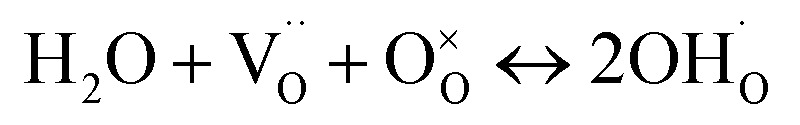
4
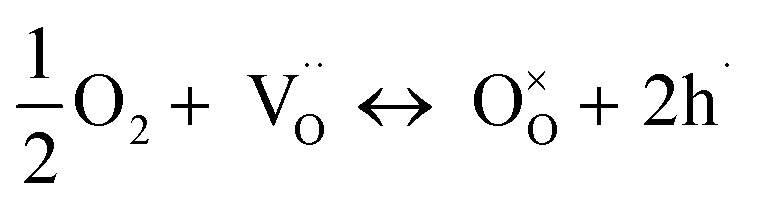


The relationship between the partial conductivities of protons, oxide ions, holes, and atmosphere can be calculated by equilibrium constant (eqn (3) and (4)):5
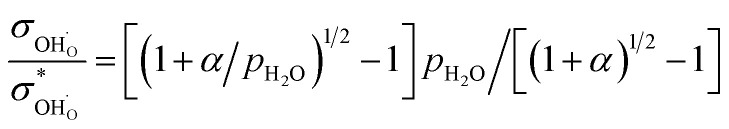
6
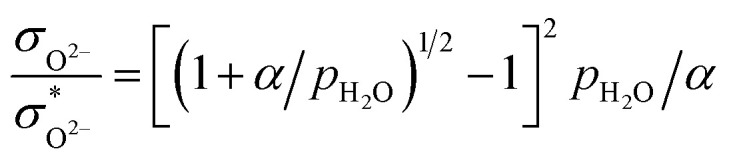
7
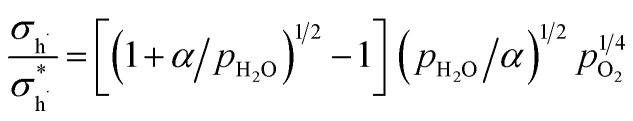
where 
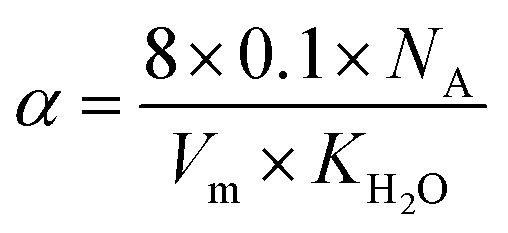
, and *N*_A_, *V*_m_, *K*_H_2_O_ are Avogadro's number, the molar volume of the system, the equilibrium constant of eqn (3), and 
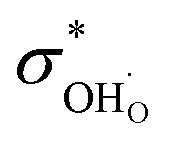
, 
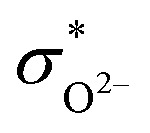
, and 
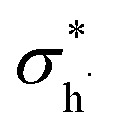
 are the conductivities of proton in *p*_H_2_O_ = 1 atm, oxide ion in *p*_H_2_O_ = 0 atm, and hole in *p*_O_2__ = 1 atm and *p*_H_2_O_ = 0 atm, respectively.

Transport numbers can be expressed as follows:8
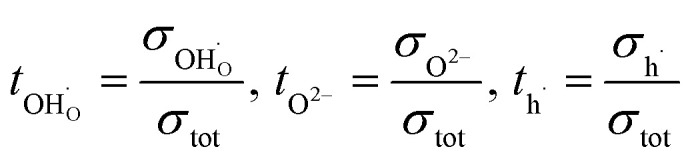


The proton mobility 
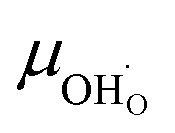
 and oxide ion mobility *μ*_O^2−^_ can be calculated as follows:9
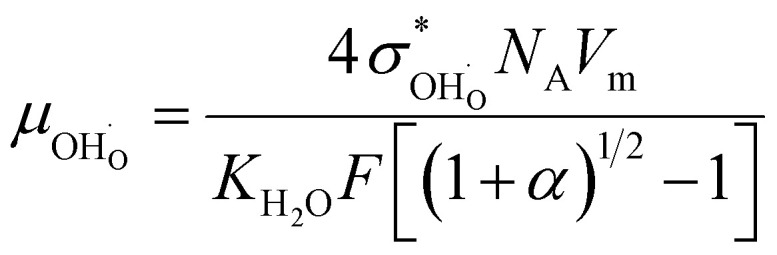
10
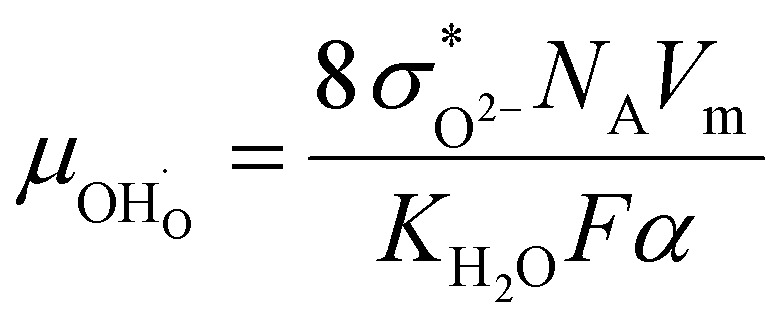
where *F* is Faraday's number.

The proton concentration 
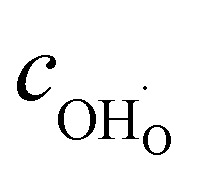
 and oxide ion concentration *c*_O^2−^_ can be calculated as follows:11
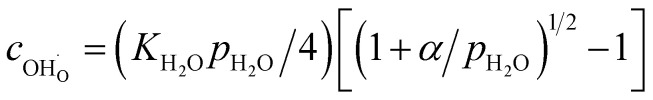
12
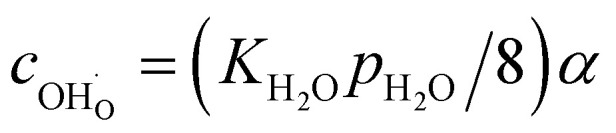


## 3. Results and discussion

### 3.1 Phase composition and structural analysis

**Fig. 1:**
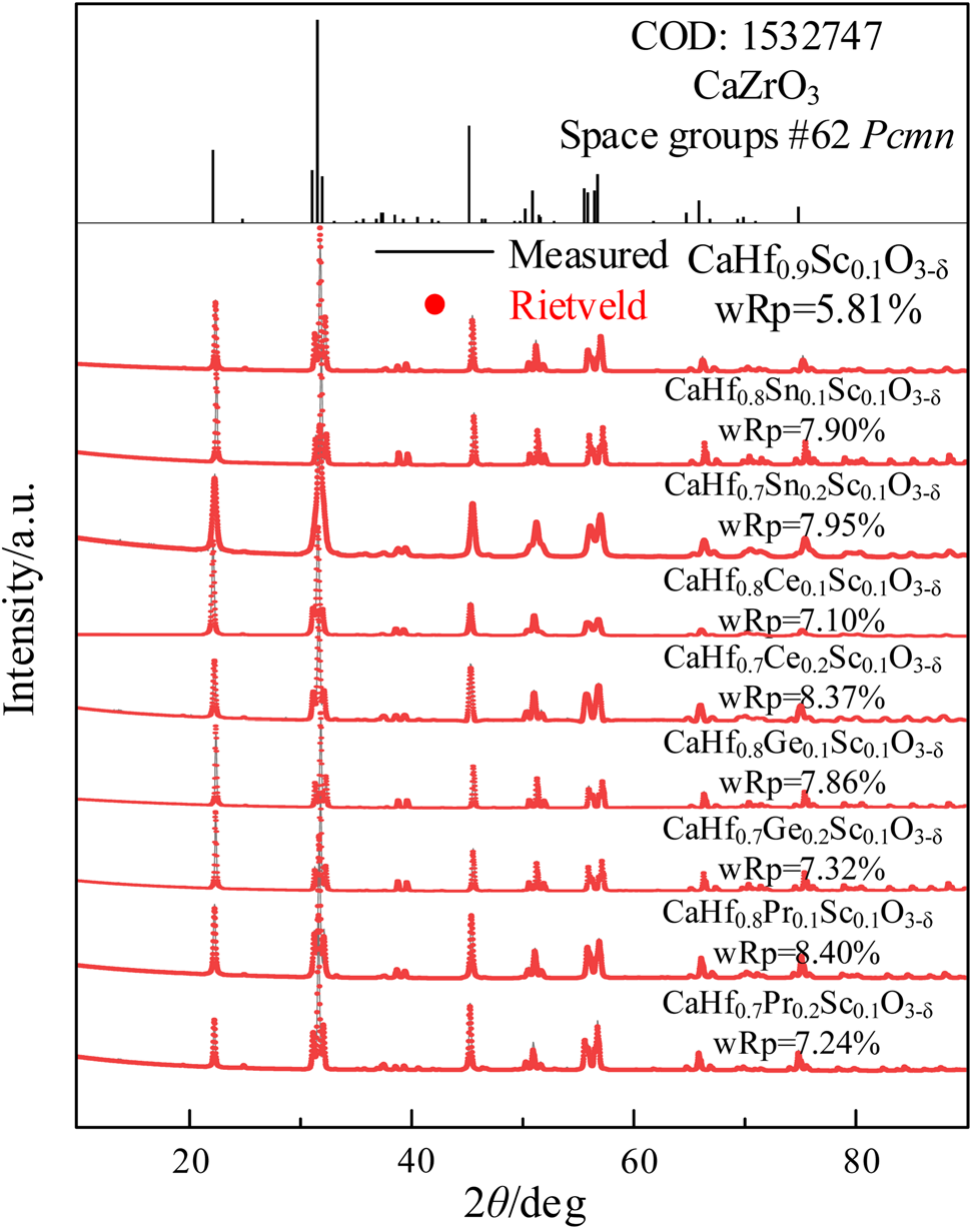
XRD patterns of CHMSs sintered at 1600 °C for 10 h in air.

The XRD patterns of CHMSs prepared at 1400 °C for 10 h and 1600 °C for 10 h under air atmosphere are shown in Fig. 1, with the crystal parameters are listed in Table 1. Due to the lack of standard CIF files for CaHfO_3_ crystals, CaZrO_3_ (ref. 23) was utilized as a reference. Obviously, all XRD patterns of CHMSs closely resembled to those in CaZrO_3_, which can be ascribed to the similar average ionic radii of B-site ions of CHMSs (0.678–0.746 Å) and Zr^4+^ (0.71 Å). Also, no impurity peaks were observed, indicating successfully synthesized CHMSs. The crystal parameters of sintered CHMSs, obtained by Rietveld refinement (Highscore) revealed w*R*_p_s values lower than 8.5%. The presence of fewer atoms in CHMSs compared to CaZrO_3_, due to oxygen vacancies, resulted in smaller unit cell volumes for the doped samples. Meanwhile, the unit cell volumes increased with average ionic radii of B-site ions (Ge 0.53 Å < Sn 0.69 Å < Hf 0.71 Å < Pr 0.85 Å < Ce 0.87 Å).

**Table 1:** Lattice parameters, lattice volumes, tolerance factors, and octahedral tilting of CHMSs

Material	*a* (Å)	*b* (Å)	*c* (Å)	Unit cell volume (Å^3^)	Tolerance factor	Octahedra tilting (°)
CaZrO_3_	5.583	8.007	5.759	257.45	0.912	16.08
CaHf_0.9_Sc_0.1_O_3−*δ*_	5.719	7.979	5.572	254.26	0.915	15.76
CaHf_0.8_Ge_0.1_Sc_0.1_O_3−*δ*_	5.562	7.978	5.731	254.31	0.923	14.92
CaHf_0.7_Ge_0.2_Sc_0.1_O_3−*δ*_	5.733	7.980	5.564	254.55	0.931	14.07
CaHf_0.8_Sn_0.1_Sc_0.1_O_3−*δ*_	5.723	7.977	5.573	254.42	0.916	15.66
CaHf_0.7_Sn_0.2_Sc_0.1_O_3−*δ*_	5.707	7.964	5.567	253.02	0.917	15.55
CaHf_0.8_Pr_0.1_Sc_0.1_O_3−*δ*_	5.732	8.000	5.582	255.97	0.909	16.40
CaHf_0.7_Pr_0.2_Sc_0.1_O_3−*δ*_	5.764	8.028	5.601	259.18	0.903	17.04
CaHf_0.8_Ce_0.1_Sc_0.1_O_3−*δ*_	5.739	8.001	5.583	256.36	0.908	16.51
CaHf_0.7_Ce_0.2_Sc_0.1_O_3−*δ*_	5.758	8.012	5.592	257.98	0.901	17.25

The tolerance factors, which indicate the distortion of the ABX_3_ lattice, can be calculated using eqn (2). However, the tolerance factor alone does not adequately represent the distortion associated with the tilting angle of the BO_6_ octahedra. For example, O'Keeffe^24^ calculated this tilting angle using eqn (13).13
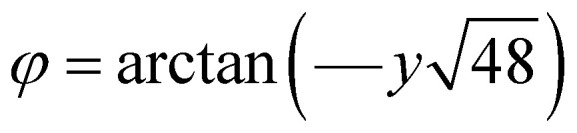
where *y* represents the fractional coordinate *y* of O′′ in 
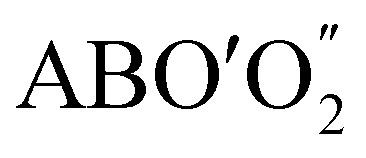
-type oxide with *Pnma* space group.

**Fig. 2:**
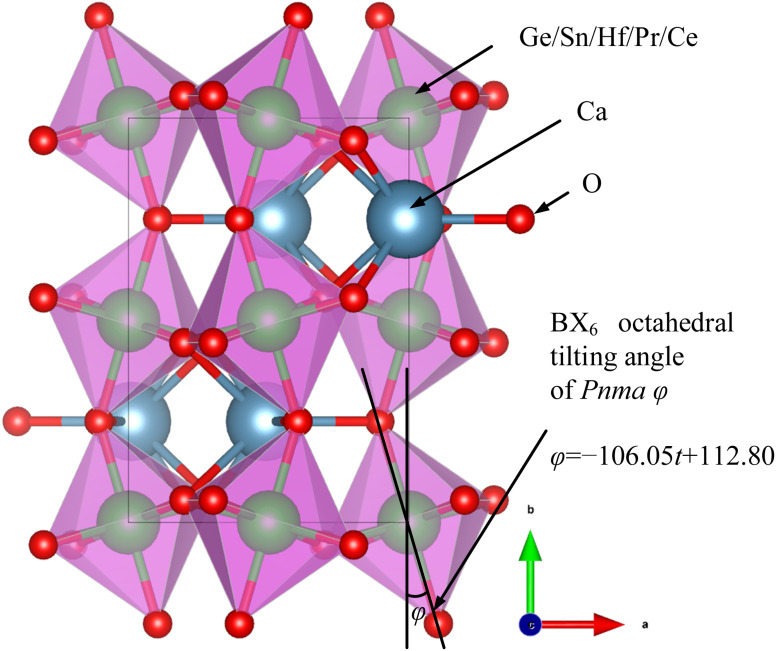
The relationship between BX_6_ octahedral tilting angle and crystal structure.

**Fig. 3:**
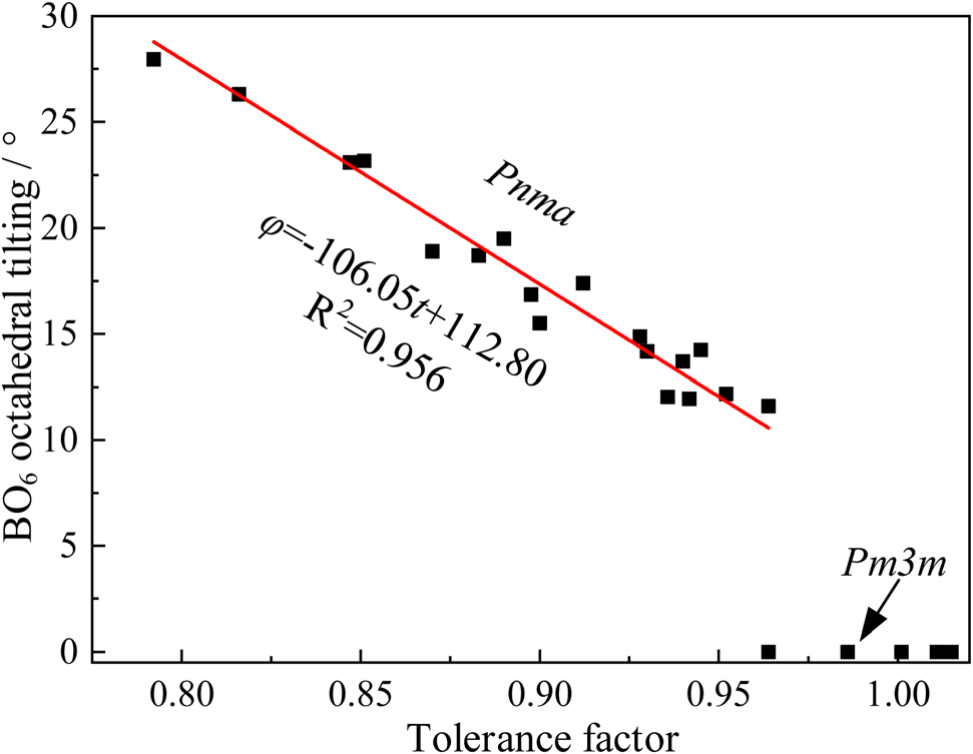
The dependence of BO_6_ octahedral tilting on tolerance factor.

However, numerous errors may be present in the calculation of fractional coordinates *via* XRD and Rietveld refinement. Additionally, doped perovskites often contain more than two types of BO_6_ octahedra, making the calculation of the tilting angle using this method challenging. Consequently, our study utilized CIF files of undoped ABX_3_-type oxides and halides from the Crystallography Open Database (COD). The angles between the BX_6_ octahedra and the lattice were measured using a geometric method in the crystal visualization software VESTA^25^ (Fig. 2). The relationship between the BO_6_ octahedral tilting and tolerance factor, depicted in Fig. 3, showed a linear increase in BX_6_ octahedral tilting as the tolerance factor decreased from 0.95 to 0.80. Notably, the crystal structure transitions to a cubic form when the tolerance factor exceeds 0.95. From this analysis, an equation was derived through curve fitting (eqn (14)):

14 *φ* = −106.05*t* + 112.80

Accordingly, the proposed perovskites, such as CHPS20 possessed HfO_6_, PrO_6_, and ScO_6_ types BO_6_ octahedra, with tilting angles of 15.61°, 21.61°, and 17.19°, respectively. The average tilting angle of CHPS20 estimated by eqn (10) was 17.04°.

### 3.2 X-ray photoelectron spectroscopy

**Fig. 4:**
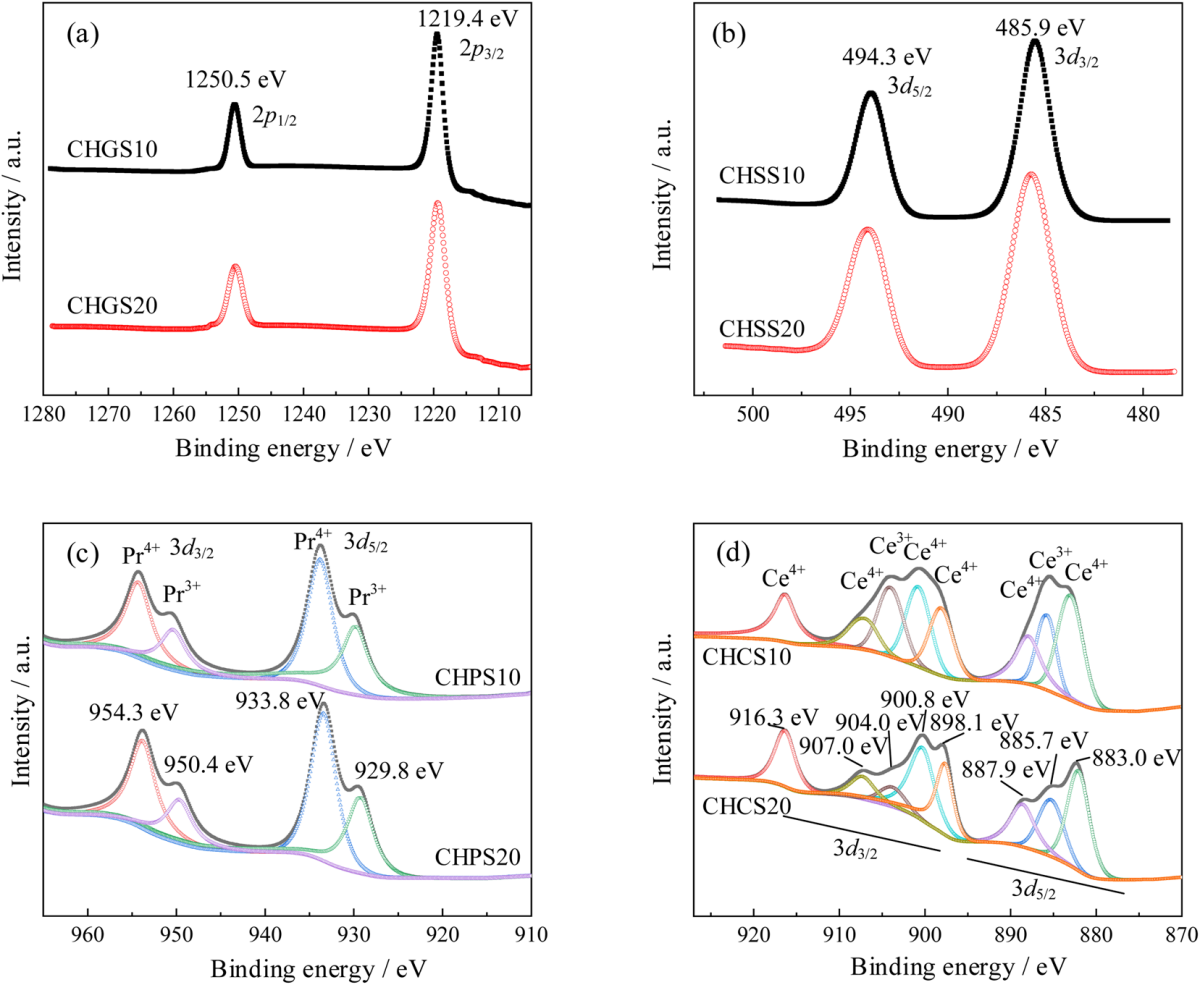
(a) Ge 2p, (b) Sn 3d, (c) Pr 3d, and (d) Ce 3d XPS spectra of CHMSs.

To determine the valence states and oxygen vacancy concentration in CHMSs, spectra of Ge 2p, Sn 3d, Ce 3d, and Pr 3d were obtained and shown in Fig. 4. The binding energy was calibrated using the C 1s peak at 284.8 eV. In high-resolution XPS spectra, both the Ge 2p and Sn 3d signals were each split into two peaks corresponding to Ge 2p_1/2_, Ge 2p_3/2_, Sn 3d_5/2_, and Sn 3d_3/2_, respectively.^26,27^ Peaks which represent Ge^2+^ and Sn^2+^ were be observed, indicating that the incorporation of Ge and Sn do not alter the oxygen vacancy concentration. Fig. 4(c) shows the Pr 3d_5/2_ and Pr 3d_3/2_ XPS spectra, where four peaks representing Pr^4+^ and Pr^3+^ were clearly discernible.^28^ The valence reduction of Pr^4+^ can be estimated by calculating the area ratio of the Pr^3+^ peaks to Pr^4+^ peaks. The ratio values of Pr^4+^ to Pr^3+^ were 1.17 in CHPS10, and 1.35 in CHPS20, suggesting that the tendency for valence reduction decreases with increased Pr doping to maintain charge neutrality. Thus, the chemical formulas of CHPSs were determined to be CaHf_0.8_Pr_0.1_Sc_0.1_O_2.927_ (Pr^4+^ = 0.054, Pr^3+^ = 0.046) and CaHf_0.7_Pr_0.2_Sc_0.1_O_2.908_ (Pr^4+^ = 0.115, Pr^3+^ = 0.085), respectively. Fig. 4(d) shows the Ce 3d_5/2_ and Ce 3d_3/2_ XPS spectra,^29,30^ the ratio values of Ce^4+^ to Ce^3+^ were 3.15 in CHCS10, and 4.68 in CHCS20, indicating a similar decrease in the valence reduction tendency with increased Ce doping. Thus, the chemical formulas of CHCSs were determined to be CaHf_0.8_Ce_0.1_Sc_0.1_O_2.938_ (Ce^4+^ = 0.076, Ce^3+^ = 0.024) and CaHf_0.7_Ce_0.2_Sc_0.1_O_2.933_ (Ce^4+^ = 0.165, Ce^3+^ = 0.035), respectively.

### 3.3 Microstructural characterization

**Fig. 5:**
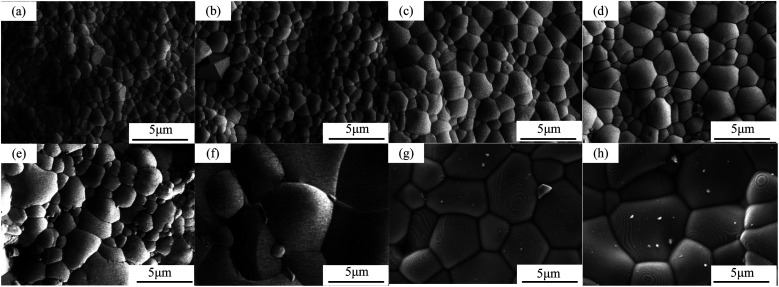
FESEM images of surface CHMSs: (a and b) M Ge, (c and d) M Sn, (e and f) M Pr, and (g and h) M Ce. (a, c, e, g) *x* = 0.1, (b, d, f, h) *x* = 0.2.

The surface morphologies of sintered CHMSs, as depicted in Fig. 5, exhibited dense morphologies devoid of any pores. The densities were measured using the Archimedes method, and the theoretical densities were calculated by atomic weights in unit cells and unit cell volumes (Table 1). Notably, all relative densities were above 98%. Interestingly, the grain size increased linearly with the average ionic radius at the B-site. This phenomenon has been previously observed in other materials, such as BaCeO_3_,^31,32^ although an inverse trend has been reported in BaZrO_3_ proton conductors.^33^ Consequently, it can be deduced that there is no direct correlation between ionic radius and grain size. In the context of refractories, Dhuban and Erkalfa *et al.*^34,35^ have reported that changes in valence can activate lattice, enhancing grain boundary mobility and atomic movement during sintering. In this study, we consider this trend from the perspective of variable valence. The calculated Gibbs free energy for the valence reduction of Ge^4+^, Sn^4+^, Pr^4+^, and Ce^4+^, derived from standard thermodynamic data of inorganic compounds, revealed that the valence reduction tendencies of these ions are similar to their ionic radii. Notably, the valence reduction tendencies of Pr^4+^ and Ce^4+^ were significantly higher than those of Ge^4+^ and Sn^4+^. Additionally, the XPS analysis results presented in Section 3.2 support this trend. Iguchi *et al.*^36^ reported a slight enrichment of dopants at grain boundaries, with a higher equilibrium concentration of oxygen vacancies than in the grain interior. Therefore, this trend can be explained by the enrichment of oxygen vacancies at grain boundaries, which facilitates the necessary migration pathways for mass transport during the sintering process, thereby promoting grain growth.

### 3.4 Electrochemical impedance spectroscopy

**Fig. 6:**
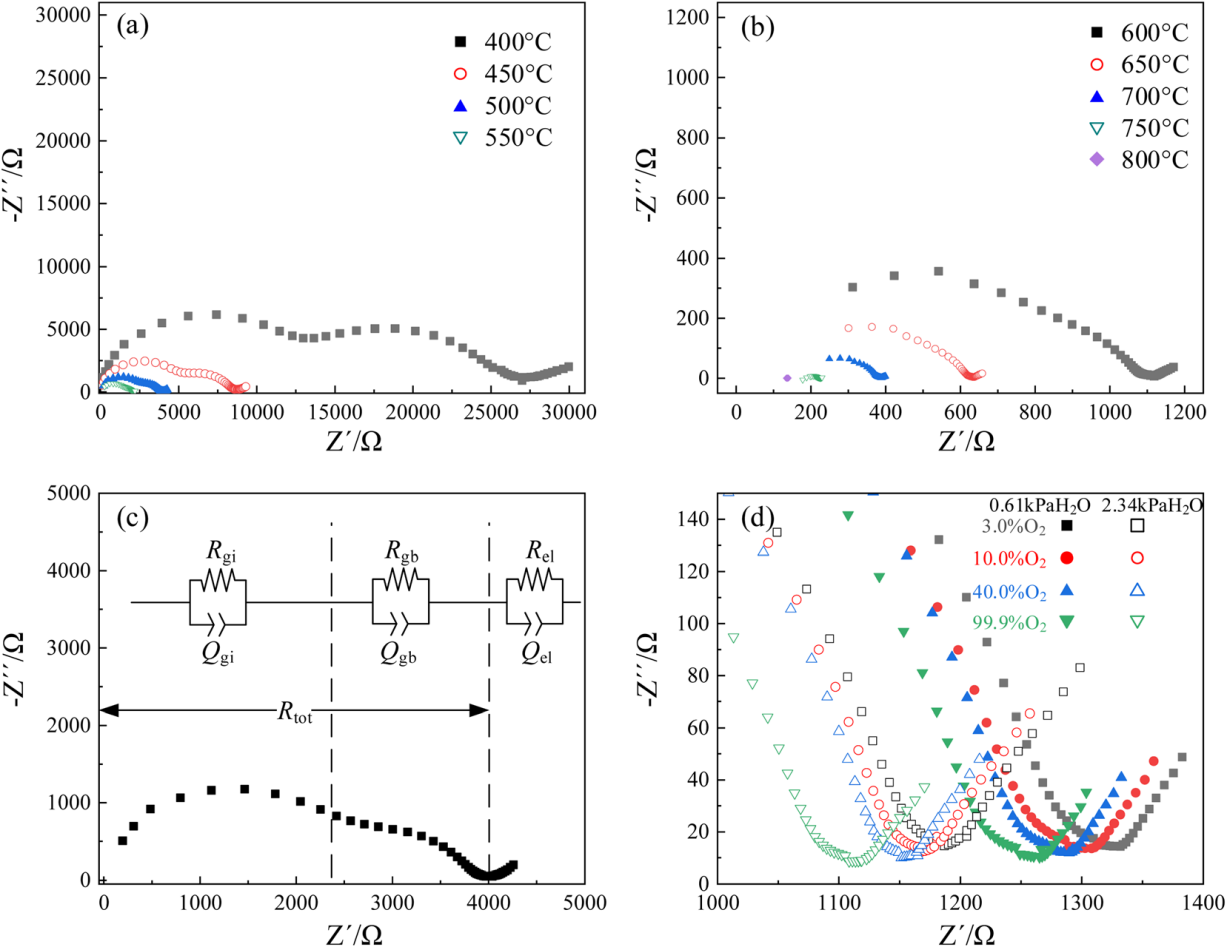
Nyquist plots of CHSS10: (a and b) under an atmosphere of 99.9% O_2_ and 2.34 kPa H_2_O at 400–800 °C, (c) at 500 °C, and (d) low-frequency parts *versus* different atmospheres at 600 °C.

Nyquist plots of CHMSs were obtained by impedance spectroscopy, with those for CHSS10 displayed in Fig. 6. Notable transformations were observed in the impedance semicircles, necessitating the use of a constant phase angle element (*Q*) in place of capacitance (*C*). Note that Gi, gb, and el represent grain interior, grain boundary, and electrode or interface process, respectively. These three components were easily distinguishable and well-fitted with equivalent circuit at 400–600 °C. The impedance of the grain boundary decreased rapidly with increasing temperature, a trend not evident in the impedance of the grain interior at 600–800 °C. Hence, total conduction at these temperatures was dominated by the conduction of grain boundary.

Fig. 6(d) shows the low-frequency region of Nyquist plots *versus* different atmospheres at 600 °C. As oxygen and vapor partial pressure rose, total resistance decreased with the lowest resistance observed under 2.34 kPa H_2_O and 99.9% O_2_. According to eqn (3) and (4), water and oxygen molecules competed to occupy the oxygen vacancies under wet atmosphere containing oxygen.^37^ Oxygen vacancies provided sites for the transport position of oxide ions and hydration, while oxidation decrease the concentration of oxygen vacancies and limit the oxide ionic conduction.

As a result, the oxide ionic transport number was always below 0.1 under similar wet atmosphere containing oxygen. Additionally, water increases the concentration of protons, while oxygen enhances the concentration of holes. Here, total resistances decreased by 11% with a 1.71% increase in water vapor, and resistance decreased by 6.2% with a 97% increase in water vapor. Thus, water molecules preferentially occupy oxygen vacancies, generating a higher concentration of protons than holes. Since mobility of proton was also higher than that of hole in ABO_3_-type proton conductor,^37,38^ protonic conduction likely dominates at 600 °C.

### 3.5 Conductivity and transport numbers

**Fig. 7:**
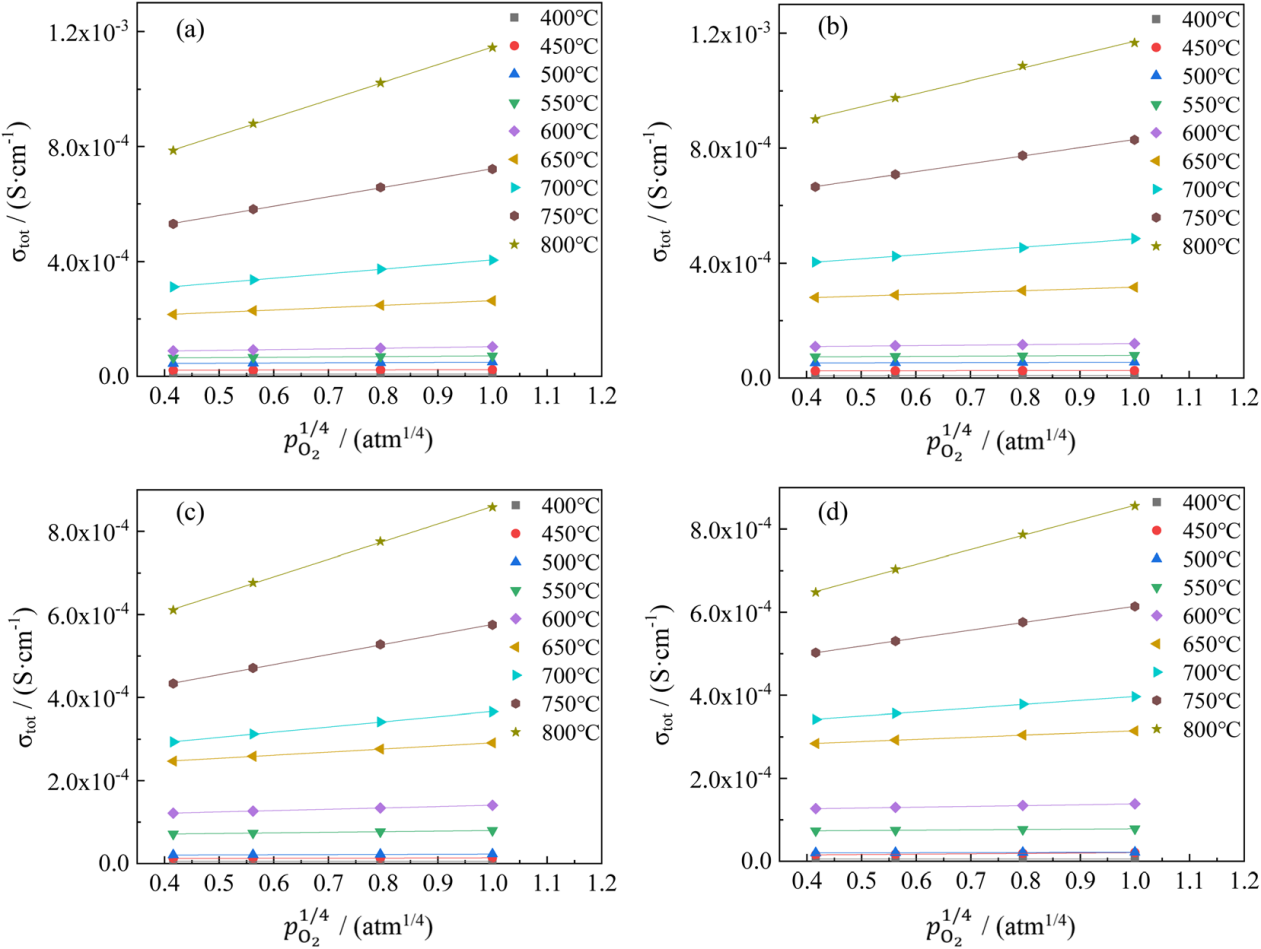
The conductivities of CaHf_0.9−*x*_Sn_*x*_Sc_0.1_O_3−*δ*_*versus* oxygen partial pressure under different vapor partial pressures: (a) (*x* = 0.1, *p*_H_2_O_ = 0.61 kPa), (b) (*x* = 0.1, *p*_H_2_O_ = 2.34 kPa), (c) (*x* = 0.2, *p*_H_2_O_ = 0.61 kPa), and (d) (*x* = 0.2, *p*_H_2_O_ = 2.34 kPa).

The changes in total conductivities of CHSSs *versus*
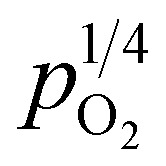
 under different vapor partial pressures are provided in Fig. 7. All conductivities presented a linear increase as a function of 
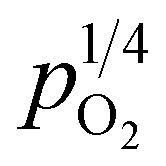
, a relationship that can well be fitted with eqn (4). This indicates that the defect equilibria model was suitable for CHSSs, and a similar relationship is observed in all CHMSs. While some studies have noted a decrease in slope of conductivity *versus*
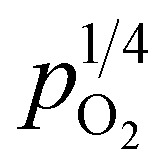
 with increasing vapor partial pressure, opposite behavior was documented in our previous studies.^38,39^

In general, such behavior is commonly observed in ABO_3_-type proton conductors, where the slope tends to increase in double-type proton conductors. This phenomenon may be attributed to the rise in protonic conductivity combined with a decline in oxide ionic conductivity as a function of vapor partial pressure (eqn (3)). The difference change in these conductivities determines the slope of conductivity *versus*
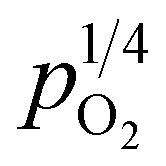
. Therefore, the slope decreased with high protonic transport number, while it increases in proton conductors with significant oxide ionic conduction. Also, the oxide ionic transport numbers in AB′B′′O_3_ are often higher than those of simple ABO_3_ due to enhanced oxygen vacancy concentration tolerance in AB′B′′O_3_.^38,39^ In this study, all slopes decreased as a function of vapor partial pressure, likely due to high protonic transport number in CHMSs.

**Fig. 8:**
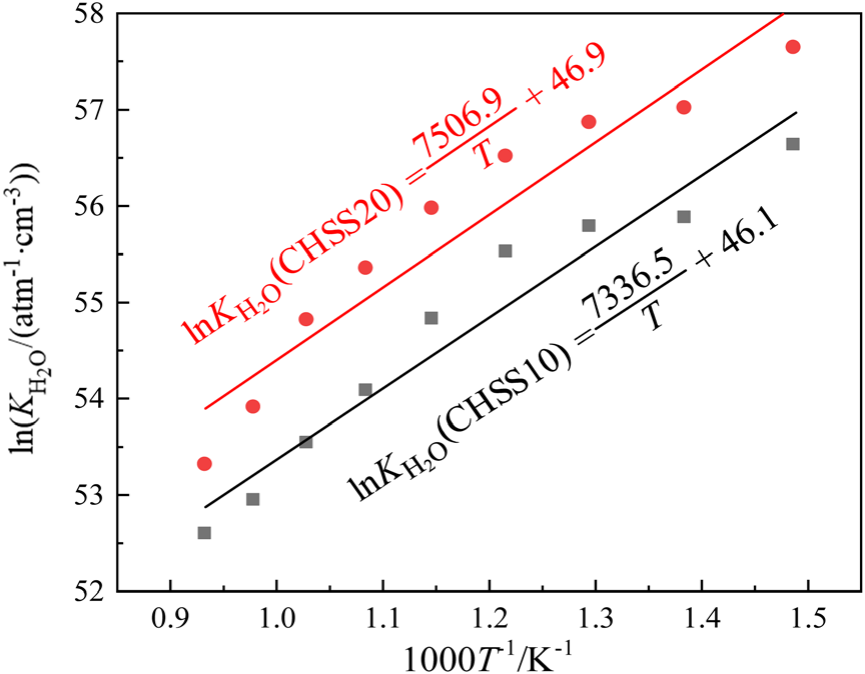
Dependence of equilibrium constant (*K*_H_2_O_) on temperature of CHSSs.

After determining the key parameter *α* of defect equilibria model in Fig. 7, the dependence of equilibrium constant (*K*_H_2_O_) values of eqn (3) on temperature of CHSSs were calculated (Fig. 8). All standard molar hydration enthalpy Δ*H*^θ^_m_ values of CHMSs were then calculated from the slope of ln *K*_H_2_O_*versus* temperature (Table 2).

**Table 2:** Conduction data of CHMSs under *p*_H_2_O_ = 2.34 kPa and 20% O_2_/Ar atmospheres

	BaZr_0.8_Y_0.2_O_3−*δ*_ (ref. 40)	BaCe_0.9_Y_0.1_O_3−*δ*_ (ref. 41)	CHGS20	CHGS10	CHSS20	CHSS10	CHPS10	CHCS10	CHPS20	CHCS20
*σ*_tot_ (800 °C)/S cm^−1^	∼5 × 10^−2^ (700 °C)	∼5 × 10^−2^	1.20 × 10^−5^	1.63 × 10^−5^	8.36 × 10^−4^	1.04 × 10^−3^	6.79 × 10^−5^	8.41 × 10^−5^	6.52 × 10^−7^	6.18 × 10^−5^
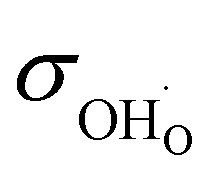 (800 °C)/S cm^−1^		∼8 × 10^−3^	5.19 × 10^−6^	7.64 × 10^−6^	3.67 × 10^−4^	4.99 × 10^−4^	3.38 × 10^−5^	4.23 × 10^−5^	2.83 × 10^−7^	2.67 × 10^−5^
*σ*_O^2−^_ (800 °C)/S cm^−1^		∼3 × 10^−2^	3.06 × 10^−6^	3.39 × 10^−6^	2.08 × 10^−4^	1.99 × 10^−4^	9.69 × 10^−6^	1.16 × 10^−5^	1.18 × 10^−7^	1.19 × 10^−5^
*σ*_h˙_ (800 °C)/S cm^−1^		∼1 × 10^−2^	3.06 × 10^−6^	5.23 × 10^−6^	2.61 × 10^−4^	3.46 × 10^−4^	2.45 × 10^−5^	3.01 × 10^−5^	2.51 × 10^−7^	2.31 × 10^−5^
Δ*H*^θ^_m_/kJ mol^−1^			−62.4	−61.0	−64.8	−61.0	−63.2	−58.9	−82.6	−77.5
*E*_tot_/eV	∼0.62	1.04	1.04	0.88	0.81	0.97	0.70	1.00	0.69	
*E*_OH˙_/eV			0.94	0.94	0.78	0.72	0.89	0.62	0.90	0.59
*E*_O^2−^_/eV			1.62	1.89	1.77	1.57	1.45	1.26	1.68	1.12
*E*_h˙_/eV			1.35	1.38	1.21	1.18	1.23	1.02	1.31	1.05
*t*_OH˙_ (800 °C)	<0.1	∼0.16	0.434	0.470	0.439	0.478	0.497	0.503	0.433	0.433
*t*_O^2−^_ (800 °C)		∼0.64	0.256	0.208	0.249	0.190	0.143	0.138	0.181	0.193
*t*_h˙_ (800 °C)	∼0.20	0.310	0.322	0.312	0.331	0.360	0.358	0.385	0.373	
*μ*_OH˙_/(cm^2^ (V^−1^ s^−1^))(800 °C)	∼9.3 × 10^−5^	∼3.1 × 10^−4^	3.12 × 10^−7^	6.15 × 10^−7^	2.03 × 10^−5^	3.84 × 10^−5^	2.95 × 10^−6^	2.13 × 10^−6^	1.59 × 10^−8^	1.58 × 10^−6^
*μ*_O^2−^_/(cm^2^ (V^−1^ s^−1^))(800 °C)	∼1.3 × 10^−7^	∼1.4 × 10^−3^	2.41 × 10^−7^	2.93 × 10^−7^	8.96 × 10^−6^	1.13 × 10^−5^	4.96 × 10^−7^	4.86 × 10^−7^	8.63 × 10^−9^	7.71 × 10^−7^
*c*_OH˙_/mol% (800 °C)		∼3.0	6.1	4.0	4.3	4.2	3.7	5.9	5.7	5.3
*c*_O^2−^_/mol% (800 °C)		∼3.5	6.9	12.1	10.2	11.6	12.7	8.1	8.7	9.4

In this study, the electrical properties are the primary focus of analysis, particularly the impact of the degree of octahedral distortion on these properties. Therefore, in Table 2, the materials are arranged differently from Table 1, being listed in the increasing order of distortion of BO_6_. The study was conducted under a common atmospheric condition (20% O_2_ and 2.34 kPa H_2_O/Ar), ensuring that all conduction properties were evaluated under the same environment. All CHMSs displayed Δ*H*^θ^_m_ values ranging from −58.9–−82.6 kJ mol^−1^. Løken *et al.*^42^ estimated Δ*H*^θ^_m_ values of CaZr_0.9_Sc_0.1_O_3−*δ*_ and CaZr_0.8_Sc_0.2_O_3−*δ*_ by TG-DSC method, and recorded −58 kJ mol^−1^ and −94 kJ mol^−1^, respectively. These values are comparable to those obtained in the current study. Note that Δ*H*^θ^_m_ represents the water absorbing power of proton conductors, which can be related to oxygen vacancy through eqn (3). The valence reduction of B-site ions can generate additional oxygen vacancies, with M^4+^ ions exhibiting higher valence reduction tendencies than Hf^4+^. Therefore, M^4+^ doped materials possessed more oxygen vacancy concentrations of CHS10, and all CHMS20 showed more negative Δ*H*^θ^_m_ values than CHMS10.

In addition, Pr^4+^ and Ce^4+^ have markedly higher valence reduction tendencies than Ge^4+^ and Sn^4+^, resulting in CHPS20 and CHCS20 having the most negative Δ*H*^θ^_m_ in Table 2. Hydration decreased the concentration of oxygen vacancy and limited the oxide ionic conduction, thereby increasing the protonic transport numbers and decreasing the oxide ionic transport numbers for CHPS20 and CHCS20 relative to other materials, considering the effects of hydration. However, more oxygen vacancy concentrations may negatively affect protonic conduction. Regarding the protonic microscopic migration pathway, proton can jump to nearest eight oxygen sites in an undoped or fully hydrated proton conductor. The number of jumpable oxygen sites decreases in proton conductors with more oxygen vacancies in the same atmosphere, thereby enhancing apparent activation energy. Therefore, the properties of CHMSs containing valence reduction elements warrant more comprehensive studies.

**Fig. 9:**
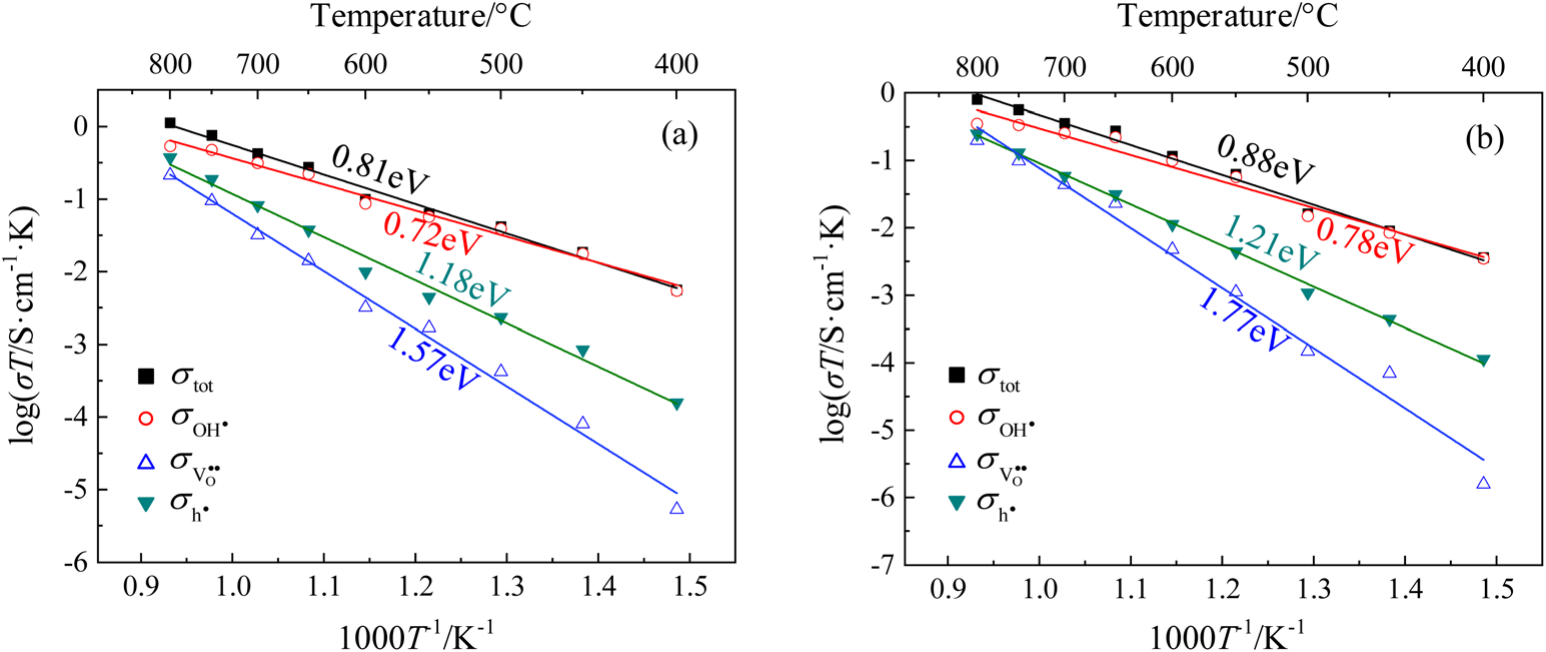
Arrhenius curves of total, protons, oxide ions, and holes for (a) CHSS10 and (b) CHSS20.

The partial conductivities of CHMSs under *p*_H_2_O_ = 2.34 kPa and 20% O_2_/Ar were calculated by defect equilibria model, with select data presented in Table 2. Meanwhile, the partial conductivities and activation energies for each carrier of CHSSs are provided in Fig. 9. According to Table 2, CHSS displayed the highest conductivities, while an excess Pr dopant significantly restricted conduction due to the surplus of oxygen vacancies. Lim *et al.*^21,22,43^ estimated typical activation energies of proton, oxide ion, and hole to be ∼0.7 eV, ∼0.9 eV, and ∼1.4 eV, respectively. BaCe_0.9_Y_0.1_O_3−*δ*_ (BCY10), like CHMSs, also possesses an orthorhombic structure with a *pnma* space group, but exhibits considerably less octahedral distortion relative to CHMSs. In contrast, the cubic structure of BaZr_0.8_Y_0.2_O_3−*δ*_ (BZY20) lacks orthorhombic distortion. Consequently, the total activation energy of BCY10 is 0.62 eV, markedly lower than typical activation energy values. Furthermore, both the conductivities and protonic mobilities of BZY20 and BCY10 surpass those of CHMSs, implying that charge carrier conduction in these materials is not impeded by their structural configurations. In our study, the activation energies for the three carriers were calculated as ∼0.8 eV, ∼1.5 eV, and ∼1.2 eV, respectively. All activation energies of oxide ions were higher than those of holes, deviating from typical values. Hence, the conduction of oxide ions was strongly limited in CHMSs. Additionally, both the conduction of proton and oxide ions were limited, as expected from the distortion of BO_6_ octahedron. The activation energies of holes were lower than typical values owing to the additional oxygen vacancies generated by valence reduction. All protonic activation energies were significantly lower than those of oxide ions and holes, indicating CHMSs dominated by protonic conduction.

**Fig. 10:**
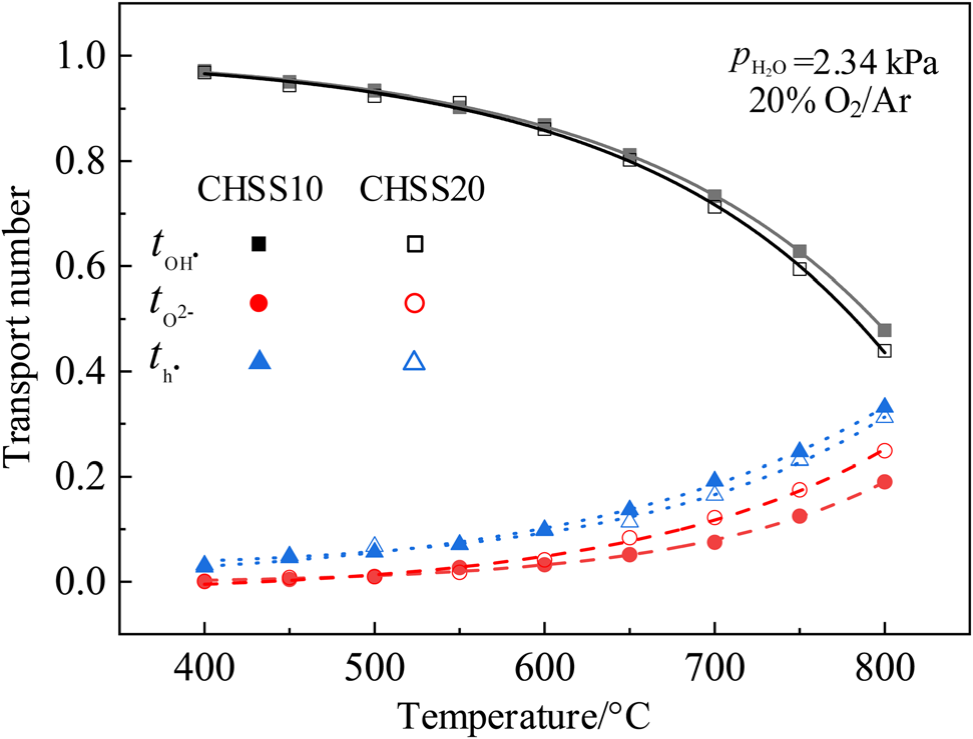
Transport numbers of proton, oxide ion, and hole for CHSSs.

Transport numbers were calculated from Fig. 9 by eqn (8), and the values of CHSSs are depicted in Fig. 10. Transport properties in Table 2 indicate that all CHMSs dominated by protonic conduction at 400–800 °C. The proton transport numbers for BZY20 and BCY10 were notably lower than those for CHMSs, while their oxide ion transport numbers were the highest. This suggests that the orthorhombic distortion in CHMSs effectively restricts oxide ion conduction. Besides, transport numbers of CHMS10 were higher than those of CHMS20, indicating limited benefit of protonic conduction by excess oxygen vacancies generated by valence reduction. CHCS10 displayed the highest protonic transport number reaching 0.961–0.503 at 400–800 °C. Protonic transport numbers of CHMS10 increased in the following order: CHGS10 < CHSS10 < CHPS10 < CHCS10. Also, oxide ionic transport numbers of CHMS10 decreased in the same order. Both trends were closely aligned with the distortion degree of BO_6_ octahedron. Transport numbers of holes for CHMSs appeared similar, with slight increasing tendency following valence reduction. Thus, the distortion degree of BO_6_ octahedron effectively enhances protonic transport number.

**Fig. 11:**
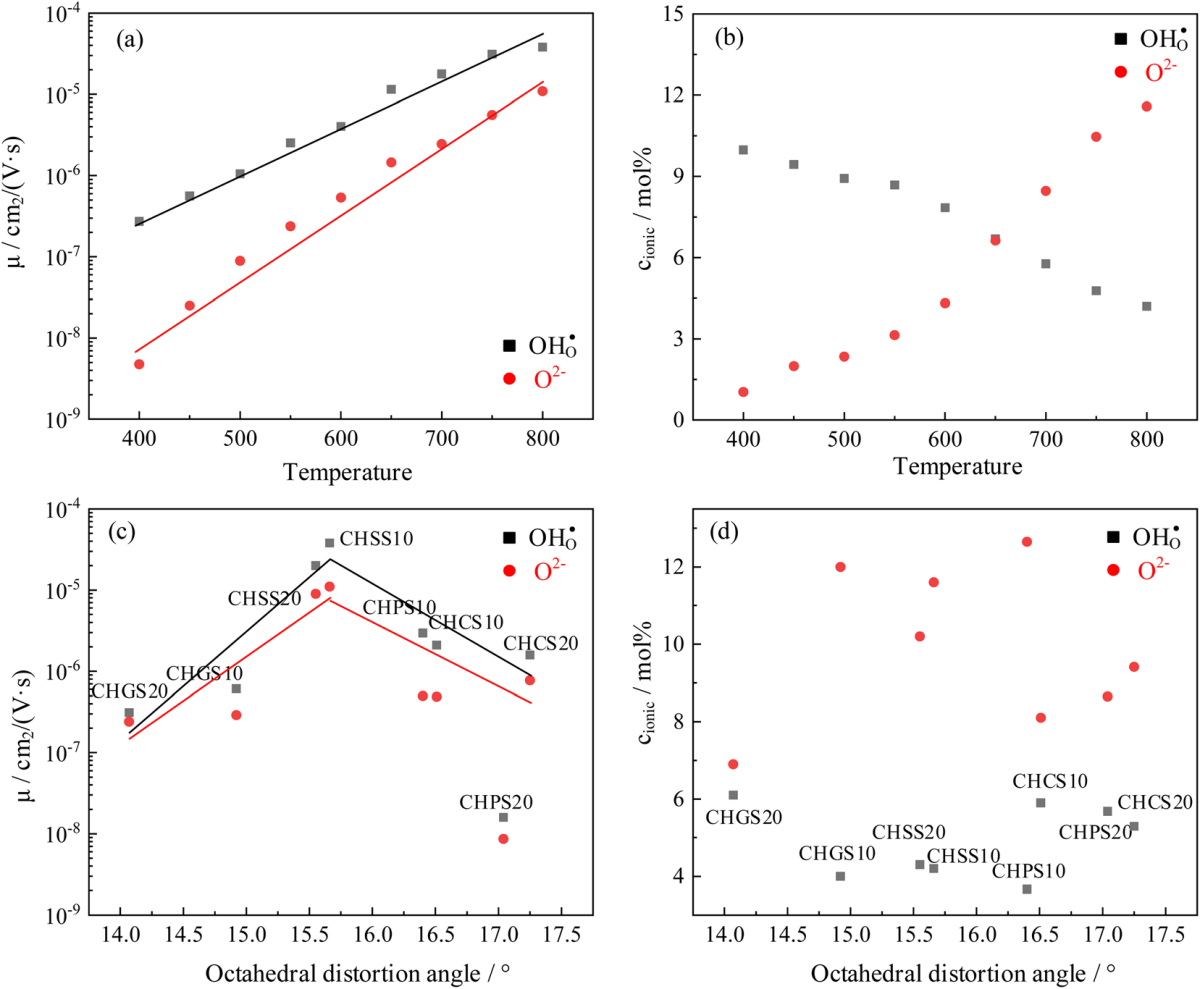
Ionic mobility and concentration of CHSSs: dependence of ionic (a) mobility and (b) concentration on temperature, dependence of ionic (c) mobility and (d) concentration on octahedral distortion angle.

It is worth noting that the transport number is a composite value, integrating various conductivities, and may not precisely elucidate the impact of distortion on distinct charge carriers. To further analyze the impact of octahedral distortion on proton and oxygen ion migration, the mobility and carrier concentration of both proton and oxygen ion were calculated (Table 2). As shown in Fig. 11(a), the logarithm of mobility exhibits a linear relationship with temperature. As temperature increases, ions acquire greater kinetic energy, resulting in increased mobility for both proton and oxygen ion. However, elevated temperatures adversely affect hydration (Fig. 8), leading to a gradual decrease in proton concentration with increasing temperature. Additionally, according to eqn (3), weakened hydration also results in a higher concentration of oxygen vacancies in the proton conductor (Fig. 11(b)). Fig. 11(c) illustrates the effect of octahedral distortion angle on proton conductor mobility. Notably, CHPS20 exhibits significantly different mobility compared to other materials, with both proton and oxygen ion being significantly suppression. We hypothesize that the excessive oxygen vacancies in CHPS20 may decrease the number of jumpable oxygen paths, thereby reducing the ionic mobility, which explains why CHPS20 does not align with the behavior of other materials. Interestingly, Fig. 11(c) shows that both proton and oxygen ion mobility initially increase with octahedral distortion angle and then decrease. The optimal octahedral distortion angle for ionic mobility is 15.6°, corresponding to the highest conductivity observed in CHSS10. Fig. 11(d) demonstrates that proton and oxygen ion concentrations remain unaffected by octahedral distortion; at 800 °C, the oxygen ion concentration in all materials exceeds the proton concentration. Overall, octahedral distortion affects ion mobility, ultimately influencing the conductivity of proton conductor.

## 4. Conclusions

CaHf_0.9−*x*_M_*x*_Sc_0.1_O_3−*δ*_ (M = Ge, Sn, Pr, and Ce; *x* = 0.1, 0.2) proton conductors were successfully prepared by solid-state reaction process. Structural analysis enabled the extraction of the dependence of BO_6_ octahedral tilting on tolerance factor. The AC impendence spectroscopy of CHMSs was studied under various oxygen and vapor partial pressures, with partial conductivities and transport numbers determined using defect equilibria model. Among the prepared CHMSs, CHSS10 displayed highest conductivity (1.04 × 10^−3^ S cm^−1^) at 800 °C, while the presence of excess Pr dopant significantly impeded conduction. The standard molar hydration enthalpies ranged from −58.9–−82.6 kJ mol^−1^, and Δ*H*^θ^_m_ demonstrated an increasing negative trend as a function of the valence reduction of B-site ions. The activation energies of protons, oxide ions, and holes were estimated to be ∼0.8 eV, ∼1.5 eV, and ∼1.2 eV, respectively. The excess oxygen vacancy generated by valence reduction limited the advantage of protonic conduction. As expected, protonic transport numbers of CHMS10 increased with the distortion degree of BO_6_ octahedron. CHCS10 exhibited the highest protonic transport number of 0.503 at 800 °C. Both proton and oxygen ion mobility initially increase with octahedral distortion angle and then decrease, and the optimal octahedral distortion angle for ionic mobility is 15.6°. In sum, insights into the relationship between the conductive property and crystal structure were provided, offering potential for the regulation of various carriers in ceramic materials through sublattice distortion to enhance proton conductivity.

## Data availability

All data generated or analyzed during this study are included in this published article.

## Conflicts of interest

The authors declare no conflict of interest.
